# Scavenger receptor class B type I knockout mice develop extensive diet-induced coronary artery atherosclerosis in an age-dependent manner

**DOI:** 10.1371/journal.pone.0318118

**Published:** 2025-05-22

**Authors:** Samuel K. Lee, Ting Xiong, Alexander S. Qian, Jeong-Ah Yoo, B. Sumayyah H. Sokeechand, Mark T. Fuller, Peter L. Gross, Richard C. Austin, Suleiman A. Igdoura, Bernardo L. Trigatti

**Affiliations:** 1 Thrombosis and Atherosclerosis Research Institute, McMaster University, and Hamilton Health Sciences, Hamilton, Ontario, Canada; 2 Department of Biochemistry and Biomedical Sciences, McMaster University, Hamilton, Ontario, Canada; 3 Centre for Metabolism, Obesity and Diabetes Research, McMaster University, Hamilton, Ontario, Canada; 4 Department of Medicine, McMaster University, Hamilton, Ontario, Canada; 5 Division of Nephrology, Department of Medicine, The Research Institute of St Joe’s Hamilton and the Hamilton Centre for Kidney Research, McMaster University, Hamilton, Ontario, Canada; 6 Department of Biology and Department of Pathology and Molecular Medicine, McMaster University, Hamilton, Ontario, Canada; University Hospital Zurich: UniversitatsSpital Zurich, SWITZERLAND

## Abstract

**Objective:**

Homozygous knockout of scavenger receptor class B type I (SR-B1) in mice with atherogenic mutations (such as knockout of the apolipoprotein E or low density lipoprotein receptor genes) results in spontaneous or diet-induced coronary heart disease characterized by atherosclerosis development in the aortic sinus and coronary arteries, platelet accumulation in coronary artery plaques, myocardial fibrosis, and early death. However, the extent of coronary artery atherothrombosis and myocardial fibrosis in mice lacking SR-B1 alone (homozygous SR-B1 knockout mice) has not been examined. Although age is a major risk factor for coronary artery disease, few studies directly examine the effects of age on susceptibility to atherosclerosis or coronary artery atherothrombosis and myocardial fibrosis in mice. Therefore, we set out to examine the effects of age on diet-induced atherosclerosis in female homozygous SR-B1 knockout mice.

**Approach and results:**

SR-B1 knockout mice exhibited little-to-no aortic sinus or coronary artery atherosclerosis at 52 weeks of age, when fed a normal diet. However when fed a high-fat, high-cholesterol, cholate-containing (HFCC) diet for 12 weeks from either 14 weeks of age (26-week-old at analysis) or 40 weeks of age (52-week-old at analysis), they developed similar degrees of atherosclerosis in their aortic sinuses. Interestingly, the older aged SR-B1 knockout mice exhibited increased coronary artery atherosclerosis, increased vascular cell adhesion molecule 1 levels and platelet accumulation in coronary arteries, and increased myocardial fibrosis and plasma levels of cardiac troponin I compared to the younger aged mice. Older-aged HFCC diet-fed SR-B1 knockout mice also exhibited reduced survival to humane endpoint. Moreover, older-aged HFCC diet-fed SR-B1 knockout mice exhibited a greater inflammatory state with increased levels of circulating interleukin-6, tumour necrosis factor alpha, and neutrophils, despite plasma lipid levels being unchanged. Consistent with the increased circulating neutrophils, older-aged HFCC diet-fed SR-B1 knockout mice exhibited increased accumulation of the neutrophil marker myeloperoxidase and increased neutrophil extracellular traps in atherosclerotic plaques in the aortic sinus and increased abundance of atherosclerotic coronary arteries containing neutrophil extracellular traps.

**Conclusions:**

HFCC diet-fed homozygous SR-B1 knockout mice develop occlusive coronary artery atherothrombosis and myocardial fibrosis in an age-dependent manner, and exhibit an increased inflammatory state with older age. Therefore, aged SR-B1 knockout mice may prove to be an attractive mouse model to analyze age-dependent mechanisms associated with coronary artery disease development, which may facilitate the discovery of more effective therapeutics to treat cardiovascular disease.

## Introduction

Coronary artery disease (CAD) is a leading cause of mortality driven by atherosclerosis development in the coronary arteries, and its prevalence and mortality significantly increase with aging [1]. Atherosclerosis is characterized by the build-up of cholesterol-rich plaque in artery walls, driven by interactions between circulating immune cells, lipoproteins, and endothelial and smooth muscle cells [2]. Endothelial cells of affected regions are activated and express high levels of adhesion receptors including vascular cell adhesion molecule 1 (VCAM-1), mediating adhesion of inflammatory cells which drive atherosclerosis development [3–6]. Rupture of mature atherosclerotic plaques or erosion of endothelial cells can trigger thrombi, either accelerating atherosclerotic plaque development or occluding the artery [7]. Thrombus formation on atherosclerotic plaques is known as atherothrombosis and can cause myocardial ischemia and infarction, commonly known as heart attack [8].

Conventional mouse models widely used for atherosclerosis research include the apolipoprotein E (ApoE) knockout (KO) and high-fat and/or high-cholesterol diet-fed low density lipoprotein receptor (LDLR) KO mice [9]. These *ApoE*^*KO/KO*^ and *Ldlr*^*KO/KO*^ mice are genetically predisposed to hypercholesterolemia and develop atherosclerosis in a spontaneous [10,11] and high-fat/high-cholesterol diet-induced manner [12,13], respectively, in the aortic sinus, the lesser curvature of the aortic arch, and other regions of large arteries where blood flow is non-laminar and with low sheer stress. However, coronary arteries in these mice are largely resistant to atherosclerosis development and hence, these mice do not present with many of the clinical complications exhibited by human CAD patients, such as myocardial infarction and early death [14].

On the other hand, genetic KO of expression or function of scavenger receptor class B type I (SR-B1) in *ApoE*^KO/KO^ or *Ldlr*^KO/KO^ mice not only accelerates spontaneous and high-fat/high-cholesterol diet-induced atherosclerosis development in the aortic sinus, but triggers the development of occlusive coronary artery atherosclerosis accompanied by fatal myocardial infarction [15–22]. This has been shown to be the result of a deficiency of SR-B1 in the liver [23]. SR-B1 is a high-affinity high-density lipoprotein (HDL) receptor expressed most abundantly in the liver and in steroidogenic tissues where it mediates the selective uptake of HDL-derived cholesterol [24,25]. Selective HDL cholesterol uptake mediated by SR-B1 in hepatocytes drives a process known as reverse cholesterol transport, in which HDL stimulates the efflux of cholesterol from peripheral tissues, including cells in the artery wall, and carries cholesterol to the liver, where HDL-cholesterol is taken up by hepatocytes and secreted into bile [24,25]. SR-B1 is also expressed at lower levels in many other cell types, including endothelial cells and macrophages, and mediates HDL-dependent atheroprotective signaling [26,27]. SR-B1 has been shown to participate in the transcytosis of both HDL and LDL across endothelial cells, a key pathway in the accumulation of lipoprotein-derived cholesterol in the artery wall [28–32]. Selective endothelial knockout of SR-B1 has been demonstrated to reduce atherosclerosis development; the opposite effect of either whole body or liver specific SR-B1 knockout [28].

Despite the important roles of SR-B1 in atherosclerosis development, only a few studies have examined diet-induced atherosclerosis in *SR-B1*^*KO/KO*^ mice [33–37]. Feeding *SR-B1*^*KO/KO*^ mice a high-fat, Western-type [35,36] or a high-fat, high-cholesterol [33] diet for a prolonged period of time (20 weeks or longer) results in small atherosclerotic plaques in the aortic sinus. On the other hand, feeding *SR-B1*^*KO/KO*^ mice a high-fat, high-cholesterol, cholate-containing (HFCC) diet results in extensive atherosclerosis development in the aortic sinus [34,37]. We have recently reported that the extent of atherosclerosis development in *SR-B1*^KO/KO^ mice fed the HFCC diet for 20 weeks is similar in the aortic sinus and in the aortas to that observed in *Ldlr*^KO/KO^ and *ApoE*^KO/KO^ mice and that atherosclerotic plaques also developed in coronary arteries, with *SR-B1*^KO/KO^ mice exhibiting about twice as much coronary artery atherosclerosis as either of the other strains [37]. CAD is commonly associated with individuals of middle- and older-age and aging is a recognized independent risk factor for CAD; despite this, most basic research studies utilize juvenile or young adult mice to study atherosclerosis. One study [38] that examined the effects of older age on atherosclerosis and associated phenotypes initiated high fat/cholesterol diet feeding when the mice were young and utilized *ApoE*^*KO/KO*^ mice, which also develop atherosclerosis spontaneously on a normal diet [10,11]. A different study examined the effects of older age on atherosclerosis and associated phenotypes in *Ldlr*^*KO/KO*^ mice [39], but these have also been shown to develop atherosclerosis spontaneously on a normal diet, albeit to a lesser degree than *ApoE* ^*KO/KO*^ mice [40]. The limitation associated with these approaches is that they may primarily reflect the length of time over which atherosclerosis has developed, rather the effects of age on susceptibility to atherosclerosis. Other studies have examined the effects of aging on high-fat and/or high-cholesterol diet fed C57BL/6J WT mice [41,42]. However, these mice have been shown to develop only low levels of atherosclerosis, making it difficult to draw clear conclusions on how aging affects susceptibility to atherosclerosis or its complications, since these mice are relatively healthy. A more suitable mouse model to study the impacts of aging on susceptibility to atherosclerosis would be one which developed little-to no atherosclerosis spontaneously when fed a normal diet, but which developed significant atherosclerosis when fed high-fat and/or high cholesterol diets. We hypothesized that *SR-B1*^*KO/KO*^ mice on a C57BL/6 background would fulfill this criterion.

To test this, we analyzed the effects of age on the susceptibility of female homozygous *SR-B1*^*KO/KO*^ mice on a C57BL/6J background (referred to hereafter as *SR-B1*^*KO/KO*^ mice) to atherosclerosis induced by a high-fat, high-cholesterol, cholate-containing diet (HFCC diet). We found very little spontaneous atherosclerosis in female *SR-B1*^*KO/KO*^ mice fed a normal diet an analyzed at 52 weeks of age, however feeding the mice the HFCC diet for 12 weeks, starting ether at 14 or 40 weeks of age induced similar sized plaques in the aortic sinus but triggered greater levels of coronary artery atherosclerosis and cardiac fibrosis older-aged *SR-B1*^*KO/KO*^ mice. Our findings suggest that *SR-B1*^*KO/KO*^ mice may be a useful model system in which to study the effect of age on susceptibility to diet induced coronary heart disease.

## Materials and methods

### Mice

All procedures involving mice were in accordance with the Canadian Council on Animal Care guidelines and were approved by the McMaster University Animal Research Ethics Board. C57BL/6J wild-type (WT) mice were originally purchased from Jackson Labs and bred in-house. *SR-B1*^*KO/KO*^ mice [43] were originally obtained from Dr. Monty Krieger (Massachusetts Institute of Technology, Cambridge, Massachusetts, USA) and crossed in-house 10+ times onto a C57BL/6J background. All mice were bred and housed in the David Braley Research Institute Animal Facility at McMaster University and had free access to normal diet (Teklad, 18% protein diet, Envigo, Madison, Wisconsin, USA) and water. Only female mice were used for this study. Any mice exhibiting malocclusion of the teeth or hydrocephalus were excluded from the study. Simple randomization was used to assign mice to study groups.

For survival studies, 14 and 40 w.o. C57BL/6J WT and *SR-B1*^*KO/KO*^ mice were placed on a HFCC diet (Teklad, TD88051, Envigo, Madison, Wisconsin, USA) containing 15% fat (7.5% from cocoa butter), 1.25% cholesterol and 0.5% sodium cholate [44] for a maximum of 20 weeks, or until they exhibited the following symptoms, which were used as criteria for humane endpoint: ruffled coat, hunched posture, lethargy, and labored breathing. A separate cohort of 40 w.o. *SR-B1*^*KO/KO*^ mice were maintained on a normal diet for survival analyses for a maximum of 20 additional weeks. Mice that reached humane endpoint or reached the end of the 20-week feeding period were humanely euthanized by CO_2_ asphyxiation while under full isoflurane anesthesia. The symptom-free survival up to 20 weeks was plotted versus time. Three of the thirteen 40 w.o. C57BL/6J mice died unexpectedly without displaying the criteria for human endpoint.indicated above.

Alternatively, mice were fed their respective diets for 12 weeks starting from either 14 weeks of age (26 w.o. at harvest and analysis) or 40 weeks of age (52 w.o. at harvest and analysis). At the end of the feeding period, mice were fasted for approximately 14 hours, then fully anesthetized with isoflurane-O_2_ in an induction chamber followed by continued anaesthesia under a nose cone. Blood was collected from fully anesthetized mice into heparinized Eppendorf tubes via cheek puncture using a lancet and used to prepare plasma. For some mice, a portion of the blood was used for flow cytometry analysis of blood cells. After blood collection, the mice were then thoracotomized and hearts and vasculature were perfused in situ through the left ventricle with phosphate-buffered saline containing 10 U of heparin/ml. Hearts were then excised and cryoprotected in 30% sucrose for 1–2 hours, then frozen above liquid nitrogen in Shandon Cryomatrix (Thermo Fisher Scientific, 6769006, Ottawa, Ontario, Canada) and stored at -80°C. Plasma was prepared by centrifugation of blood at 4,000 rpm for 5 minutes in a microcentrifuge at 4°C and stored in -80°C.

### Histology

Atherosclerosis in the aortic sinuses and coronary arteries was analyzed as previously described [17,45]. Briefly, transverse cryosections (10 μm-thick) were collected using a cryotome (Shandon Cryotome Electronic, 77210163GB, Thermo Fisher Scientific, Ottawa, Ontario, Canada) from the middle of the heart to the aortic annulus in 0.3 mm intervals (to collect cross-sections through the heart including the coronary arteries) then in 0.1 mm intervals to the top of the aortic valve leaflets (to collect sections from the aortic root). Atherosclerotic plaques were detected by oil red O staining which stains lipids, and nuclei were counter-stained with Meyer’s hematoxylin. Oil red O-stained images were captured using an Axiovert 200M microscope (Carl Zeiss Canada Ltd., Toronto, Ontario, Canada). For aortic sinus atherosclerosis analyses, cross-sectional area of atherosclerotic plaque in the section best represented by three intact valve leaflets were measured manually in a blinded manner using AxioVision software. Atherosclerosis in coronary arteries were evaluated in a blinded manner by counting the coronary arteries in 7 sections, each separated by 300 µm and covering 1800 µm up to the bottoms of the aortic valve leaflets. Coronary arteries were scored as either 0% (non-atherosclerotic - no raised atherosclerotic plaque), < 50% occluded, > 50% occluded, or 100% (fully) occluded.

Myocardial fibrosis was detected by Masson’s trichrome staining (Sigma-Aldrich, Oakville, Ontario, Canada), as previously described [17,45]. This stains collagen-rich fibrotic tissue blue/purple, and healthy myocardium red/pink [17,45]. Images for 1 transverse cryosection per mouse prior to reaching the aortic annulus (where the mitral valves start to appear) were captured using an Olympus BX41 microscope with a DP72 camera (Olympus Canada Inc., Richmond Hill, Ontario, Canada). The percentage of myocardial fibrosis was measured manually using the outline function in ImageJ software and measured as a proportion of fibrotic staining to the total area of the cross-section.

### Immunofluorescence staining

Vascular cell adhesion molecule 1 (VCAM-1) in coronary arteries was detected as previously described [17,45] using cell-culture supernatants from rat B-lymphocyte hybridoma cells that produce antibodies against mouse VCAM-1 (ATCC, CRL-1909, Manassas, Virginia, USA) [46], and AlexaFluor 594 goat anti-rat secondary antibody (Invitrogen, A-11007, Waltham, Massachusetts, USA). Nuclei were detected with 4’, 6’-diamidino-2-phenylindole (DAPI) counterstaining and all fluorescent images were captured as previously described [45] using an Olympus BX41 microscope with DP72 camera (Olympus Canada Inc., Richmond Hill, Ontario, Canada). All coronary arteries in 7 sections (covering 1800 µm apical to the bottom of the aortic annulus) were counted [45]. For VCAM-1 analysis in coronary arteries, the percentage of non-atherosclerotic coronary arteries with positive VCAM-1 staining was quantified [45]. For VCAM-1 analysis in the aortic sinus, images were captured Stellaris 5 inverted confocal microscope with a 40 × objective and a Leica SP5 camera (Leica Microsystems, Inc, Concord,ON, CA). Because all aortic sinus sections were positive for VCAM-1, the intensity of VCAM-1 staining was quantified using Image J software.

Platelets in coronary arteries were detected as previously described [17,45] using a rat anti-mouse CD41 antibody (BD Biosciences, 553847, Mississauga, Ontario, Canada) and AlexaFluor 488 goat anti-rat secondary antibody (Invitrogen, A-11006, Waltham, Massachusetts, USA). For CD41 platelet analysis, the percentage of atherosclerotic coronary arteries with positive CD41 platelet staining was quantified. Citrullinated histone H3 (Cit-H3) was detected using a rabbit anti-mouse Cit-H3 antibody (Abcam, ab281584, Waltham, Massachusetts, USA) and an AlexaFluor 488 goat anti-rabbit secondary antibody (Invitrogen, A-11008, Waltham, Massachusetts, USA). Myeloperoxidase (MPO, a marker of neutrophils) was detected using a mouse monoclonal antibody (clone 8F4; Novus Biologicals, NBP1–51148, Toronto, Ontario, Canada) using a Mouse on Mouse Immunodetection Kit (Vector Laboratories, BMK-2202, Newark, California, USA) and AlexaFluor 594-conjugated streptavidin (Thermo Fisher Scientific, S32356, Ottawa, Ontario, Canada). Nuclei were detected with DAPI. Fluorescent images of CD41/DAPI and Cit-H3/MPO/DAPI staining were captured using an Olympus BX41 microscope with DP72 camera (Olympus Canada Inc., Richmond Hill, Ontario, Canada). All coronary arteries in 7 sections (covering 1800 µm apical to the bottom of the aortic annulus) were counted as previously described [45].

For analysis of MPO staining in atherosclerotic plaques in the aortic sinus and coronary arteries, the area of the plaque positive for MPO staining was quantified relative to total plaque area and expressed as the MPO positive area as a percentage of the total plaque area. Neutrophil extracellular traps (NETs) were detected by colocalization of Cit-H3- and MPO-positive staining and the percentage of plaques positive for NETs was quantified relative to total plaque area. In addition, the number of occluded atherosclerotic coronary artery cross sections that exhibited positive NET staining was quantified relative to total number of fully occluded atherosclerotic coronary artery sections.

### Plasma analysis

Plasma alanine aminotransferase (ALT) (ALT Colorimetric Assay Kit, Cedarlane, E-BC-K235-M, Burlington, Ontario, Canada) and aspartate aminotransferase (AST) (AST Colorimetric Assay Kit, Cedarlane, E-BC-K236-M, Burlington, Ontario, Canada) were measured using the indicated commercial assay kits and following manufacturers’ instructions. Plasma total cholesterol (Cholesterol Infinity, TR13421, Thermo Fisher Scientific, Ottawa, Ontario, Canada), free cholesterol (Fujifilm Healthcare Free Cholesterol E, Cedarlane, 993–02501, Burlington, Ontario, Canada) and triglycerides (Fujifilm Healthcare L-Type Triglyceride M, Cedarlane, 998–02992, Burlington, Ontario, Canada) were measured using the indicated commercial assay kits and following manufacturers’ instructions. Esterified cholesterol levels were calculated as the difference between total cholesterol and free cholesterol measurements. Normal HDL (Fujifilm Healthcare HDL Cholesterol E, Cedarlane, 997–01301, Burlington, Ontario, Canada) was measured after using phosphotungstate-magnesium to precipitate lipoproteins other than normal HDL [47] such that the cholesterol detected after precipitation is referred to as non-precipitable HDL cholesterol as described previously [48]. Non-HDL cholesterol was calculated as the difference between total cholesterol and non-precipitable HDL cholesterol as previously described ^48^. Plasma IL-6 and TNFα were measured using commercial ELISA kits (BioLegend, 431304 and 430904, respectively, San Diego, California, USA) and following manufacturer’s protocols. Soluble vascular cell adhesion molecule 1 (sVCAM-1) and soluble intercellular adhesion molecule 1 (sICAM-1) were measured using commercial ELISA kits (R&D Systems, MVC00 and MIC100, respectively, Minneapolis, Minnesota, USA) and following manufacturer’s protocols.

### Immunoblotting

Apoliproteins (apo) A1, B (100 and 48 kDa isoforms), E, and M (26 and 21 kDa isoforms) were detected by immunoblotting as previously described [49,50]. Briefly plasma (1 μl) was subjected to sodium dodecylsulfate-polyacrylamide gel electrophoresis (SDS-PAGE) using 4–20% acrylamide gradient gels (BioRad Laboratories (Canada) Ltd., 4561096, Mississauga, Ontario, Canada). Proteins were electrophoretically transferred onto polyvinyl difluoride (PVDF) membranes (BioRad Laboratories (Canada) Ltd., 1620177, Mississauga, Ontario, Canada). Membranes were blocked overnight at 4 ºC in Tris-buffered saline (TBS: 20 mM Tris-HCL, pH 7.6, 150 mM NaCl) containing 0.1% Tween-20 (TBS-T) and either 3% bovine serum albumin (BSA; New England Biolabs, 9998S, Whitby, Ontario, Canada) or 5% skim milk powder (Carnation brand), depending on the primary antibody. Primary antisera and dilutions used were goat anti-human ApoA1 (Midland BioProducts, MBC-APA1W-G1, Nittobo America, Murrieta, CA, USA) at 1:1000 in, goat anti-ApoE (Midland BioProducts, MBC-APEX-G1, Nittobo America, Murrieta, CA, USA) at 1:4000, goat anti-ApoB (Midland BioProducts, MBC-APB-G1, Nittobo America, Murrieta, CA, USA) at 1:1000, and mouse anti-ApoM monoclonal antibody (8F12, Cell Signaling Technology, 5709S, Danvers MA, USA) at 1:1000. Blots probed for ApoA1, B and E were blocked and incubated with antibodies diluted in 3% BSA in TBS-T, and blots probed for apoM were blocked and incubated with antibody diluted in 5% skim milk in TBS-T. Blots were washed 3x in for 10 min. each in TBS-T and were then incubated with the following HRP-conjugated secondary antibodies (1:5000 in 5% skim milk in TBS-T) for 1 h at room temperature: rabbit anti-goat IgG (Jackson ImmunoResearch, 305-035-003) for ApoA1, ApoE, and ApoB, and goat anti-mouse IgG (Jackson ImmunoResearch, 115-007-003) for ApoM. Proteins were detected using enhanced chemiluminescence (ThermoFisher Scientific, PI34095, Mississauga Ontario, Canada) and quantified with a Gel Doc system (Bio-Rad Laboratories (Canada) Ltd., Mississauga, Ontario, Canada). Band intensities were normalized to the average values of samples from 14 week old C57BL6/J mice.

### Flow cytometry analysis

Flow cytometry analysis was carried out as previously described [45]. Briefly, blood was incubated with the following rat anti-mouse antibodies: FITC CD3 (BD Biosciences, 555274, Mississauga, Ontario, Canada), BV510 CD45 (Cedarlane, 103138, Burlington, Ontario, Canada), PerCP-Cyanine5.5 CD45R (B220) (eBioscience, 45-0452-80, San Diego, California, USA), PE CD11b (M1/70) (eBioscience, 12-0112-82, San Diego, California, USA), PE-Cy7 Ly6C (Cedarlane, 128018, Burlington, Ontario, Canada), PE-Dazzle-594 Ly6G (Biolegend, 127648, San Diego, California, USA), PE-Cy5 CD4 (BD Biosciences, 553050, Mississauga, Ontario, Canada), PE-Cy7 CD8a (BD Biosciences, 552877, Mississauga, Ontario, Canada), and PE NK1.1 (BD Biosciences, 553165, Mississauga, Ontario, Canada). Erythrocytes present in the blood were lysed with 1x 1-step fix/lyse solution (Thermo Fisher Scientific, 00–5333, Ottawa, Ontario, Canada). Absolute leukocyte counts were determined with the addition of 123count eBeads (Thermo Fisher Scientific, 01-1234-42, Ottawa, Ontario, Canada). Flow cytometry was performed on a BD FACSCalibur Flow Cytometry system (BD Biosciences, Mississauga, Ontario, Canada), and data were analyzed using FlowJo v10 software.

### Statistical analysis

Data was plotted and statistical analyses were carried out using Prism 10.4.1 software (GraphPad Software, Boston, Massachusetts, USA) as previously described [45]. In comparing two groups, data were subjected to the Shapiro-Wilk test for normality. Those that passed normality were analyzed by the Student’s t-test (2-tailed, unpaired), and those that failed normality were analyzed by the Mann-Whitney Rank Sum test. For multiple groups, data were analyzed by one-way ANOVA with Tukey’s Multiple Comparisons Post-Hoc test. Data with two independent variables were analyzed by two-way ANOVA with Tukey’s Multiple Comparisons Post-Hoc test. Data are presented as mean ± standard error of the mean. P values <0.05 were considered statistically significant.

## Results

### Older age increased levels of diet-induced coronary artery but not aortic sinus atherosclerosis in SR-B1 KO mice

**Fig 1:**
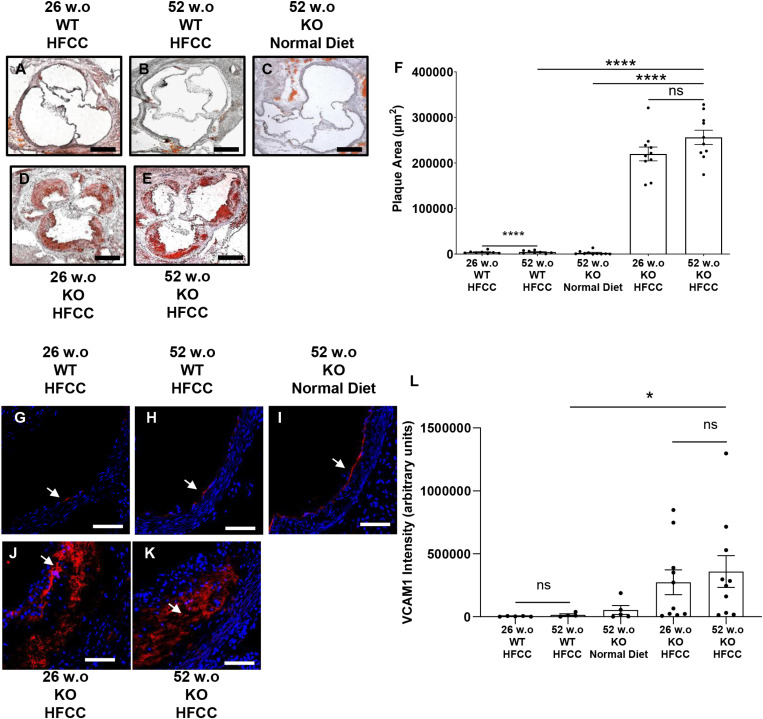
Older age did not impact the sizes or VCAM1 content of diet-induced atherosclerotic plaques in the aortic sinuses of *SR-B1*^*KO/KO*^ mice. Aortic sinus sections (representative images: oil red O and hematoxylin-stained) from **(A)** 26 w.o. and **(B)** 52 w.o. C57BL/6J (WT) mice that had been fed the HFCC diet for 12 wks; and *SR-B1*^*KO/KO*^ mice (KO) that were **(C)** 52 w.o and maintained on a normal diet, or **(D)** 26 w.o. or **(E)** 52 w.o. and had been fed the HFCC diet for 12 weeks. Scale bar = 200 μm. **(F)** Cross-sectional areas of atherosclerotic plaques in the aortic sinuses (n = 8, 8, 10, 10, 10 mice/group, respectively). Representative images of VCAM-1 immunofluorescence (red, see white arrows) and DAPI (blue) stained aortic sinus sections from **(G)** 26 w.o. and **(H)** 52 w.o. C57BL/6J (WT) mice that had been fed the HFCC diet for 12 wks; and *SR-B1*^*KO/KO*^ mice (KO) that were **(I)** 52 w.o. and maintained on a normal diet, or **(J)** 26 w.o. or **(K)** 52 w.o. and had been fed the HFCC diet for 12 weeks. Scale bar = 50 μm. **(L)** Intensity of VCAM-1 staining in each aortic sinus section. Each data point represents an individual mouse. Bars are means and error bars represent standard errors of the mean. Data in were analyzed by one-way ANOVA and Tukey’s post-hoc test. ****P < 0.0001; *P < 0.05; ns = not statistically significantly different.

Previous studies have demonstrated that C57BL/6J WT mice fed a high-fat diet develop minimal atherosclerotic lesions in the aortic sinus [41,42,44,51,52]. Consistent with these studies, 12-week HFCC diet-fed 26 w.o. and 52 w.o. C57BL/6J mice exhibited little-to-no detectable atherosclerotic plaque development in the aortic sinus (Fig 1A, 1B and F). Additionally, there were no significant histological differences in aortic sinus atherosclerosis levels between HFCC diet-fed 26 w.o. and 52 w.o. C57BL/6J mice, consistent with previous studies that examined the effects of aging on HFCC diet-fed C57BL/6J mice [41,42]. Moreover, 52 w.o. *SR-B1*^*KO/KO*^ mice on a normal diet did not develop detectable aortic sinus atherosclerosis (Fig 1C and 1F), supporting previous findings [53] that *SR-B1*^*KO/KO*^ mice develop little atherosclerosis when fed a normal diet. However, *SR-B1*^*KO/KO*^ mice fed an HFCC diet develop large atherosclerotic lesions in the aortic sinus [34,37]. Consistent with these findings, HFCC diet-fed 26 w.o. and 52 w.o. *SR-B1*^*KO/KO*^ mice developed large plaques in the aortic sinus, but there were no significant differences in plaque sizes in the older versus the younger mice (Fig 1D-1F). Immunostaining for vascular cell adhesion molecule 1 (VCAM-1) (Fig 1G-1L) revealed that all aortic sinus sections exhibited positive VCAM-1 staining. The staining in sections from the 26 and 52 w.o. C57BL/6J mice fed the HFCC diet and the 52 w.o. *SR-B1*^*KO/KO*^ mice fed the normal diet was confined to the luminal edge of the vessel, consistent with localization in endothelial cells (Fig 1G-1I). On the other hand, the majority of VCAM-1 staining in aortic sinus sections from the HFCC diet-fed 26 w.o. and 52 w.o. *SR-B1*^*KO/KO*^ mice was associated with atherosclerotic plaques (Fig 1J, 1K). Quantification of the intensity of VCAM-1 staining demonstrated increased VCAM-1 in sections from HFCC diet-fed 26 w.o. and 52 w.o. *SR-B1*^*KO/KO*^ mice compared to the 26 and 52 w.o. C57BL/6J mice fed the HFCC diet and the 52 w.o. *SR-B1*^*KO/KO*^ mice fed the normal diet (Fig 1L), reflecting the atherosclerotic plaque sizes (Fig 1F). Similar to the quantification of atherosclerotic plaque sizes, there were no differences in the abundance of VCAM1 in plaques from the HFCC diet-fed 26 w.o. and 52 w.o. *SR-B1*^*KO/KO*^ mice. Therefore, our data suggest that age does not influence the sizes or VCAM1 content of aortic sinus atherosclerotic plaques in HFCC diet-fed *SR-B1*^*KO/KO*^ mice.

**Fig 2:**
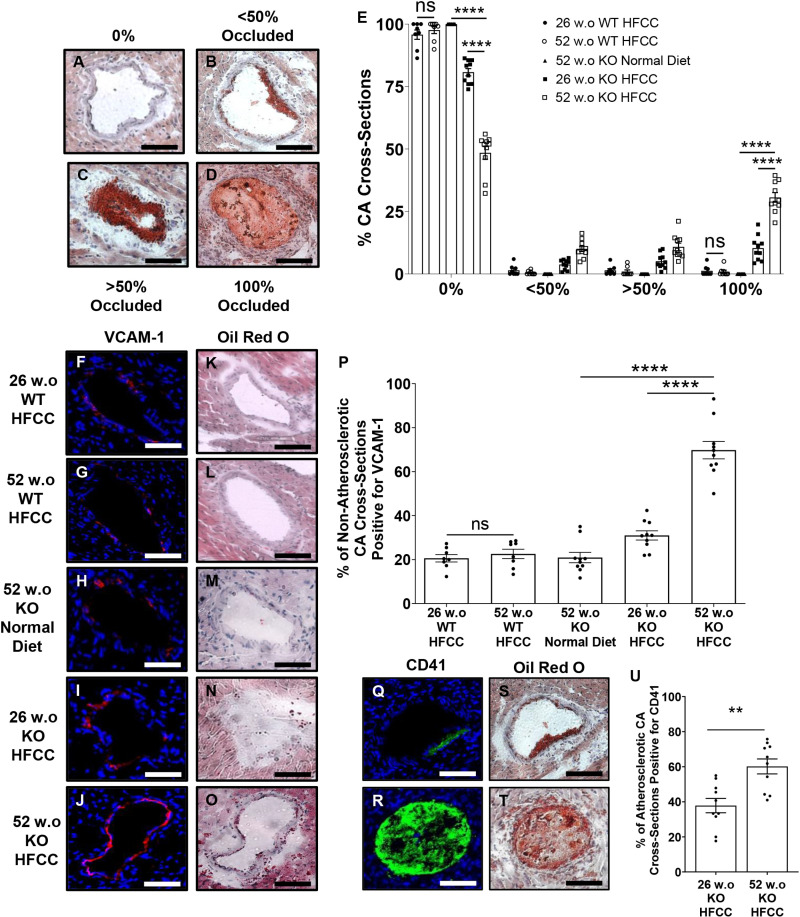
Older aged HFCC diet-fed *SR-B1*^*KO/KO*^ mice exhibit increased coronary artery atherosclerosis development, higher VCAM-1 levels in non-atherosclerotic coronary arteries and platelet levels in atherosclerotic coronary arteries. Representative images of **(A)** non-atherosclerotic (0% occluded) or **(B-D)** atherosclerotic coronary arteries with plaques occluding **(B)** <50%, **(C)** >50% or **(D)** 100% of their lumen. Scale bar = 50 μm. **(E)** Quantification of coronary arteries in each category (as percentage of total) ^17,45^ (n = 8, 8, 10, 10, 10 mice/group). Data points represent individual mice, bars represent means and error bars represent standard errors of the mean. Representative images of **(F-J)** VCAM-1 immunofluorescence (red; blue is DAPI nuclear counter-staining) and **(K-O)** oil red O and hematoxylin stained adjacent sections of non-atherosclerotic coronary artery sections from **(F, K)** 26 w.o. and **(G, L)** 52 w.o. C57BL/6J (WT) mice that had been fed the HFCC diet for 12 wks; and *SR-B1*^*KO/KO*^ mice (KO) that were **(H, M)** 52 w.o. and maintained on a normal diet, or **(I, N)** 26 w.o. or **(J, O)** 52 w.o. and had been fed the HFCC diet for 12 weeks. Representative pairs of images are shown. Scale bar = 50 μm. **(P)** Quantification of proportions of sections of non-atherosclerotic coronary arteries positive for VCAM-1 staining (n = 8, 8, 10, 10, 10 mice/group). **(Q, R)** CD41 (green)/DAPI (blue) fluorescent images and **(S, T)** oil red O/hematoxylin stained adjacent sections from **(Q, S)** 26 w.o. and **(R, T)** 52 w.o. *SR-B1*^*KO/KO*^ (KO) mice that had been fed the HFCC-diet for 12 weeks. **(U)** Quantification of the proportions of atherosclerotic coronary arteries positive for CD41 (n = 10 mice/group). For **(E)**, **(P)** and **(U)**, coronary artery cross sections were quantified across 7 histological sections of hearts from each mouse as previously described [45]. Each data point represents an individual mouse. Bars represent means and error bars represent standard errors of the mean. Data in **(E)** were analyzed by two-way ANOVA with Sidak’s multiple comparisons post-hoc test. Data in **(P)** were analyzed by one-way ANOVA and Tukey’s post-hoc test. Data in **(U)** passed the normality test and were analyzed by Student’s t-test. ****P < 0.0001; **P < 0.01; ns = not statistically significantly different.

The proportions of coronary artery cross sections exhibiting different levels of atherosclerosis (Fig 2A-2D) were quantified across 7 cardiac cross-sections from each mouse. HFCC diet-fed 26 w.o. and 52 w.o. C57BL/6J WT mice exhibited very little atherosclerotic plaque development in coronary arteries, with 95.8% ± 1.8% and 97.6% ± 1.4% of coronary artery cross-sections lacking atherosclerotic plaques (0% occluded), respectively (Fig 2E). Similarly, normal diet-fed 52 w.o. *SR-B1*^*KO/KO*^ mice exhibited no evidence of coronary artery atherosclerosis (Fig 2E). Surprisingly, despite having similar levels of atherosclerosis in the aortic sinus, 52 w.o. *SR-B1*^*KO/KO*^ mice that had been fed the HFCC diet for 12 weeks had significantly higher levels of coronary artery atherosclerosis (30.6% ± 1.9% vs. 10.4% ± 1.6% of coronary artery cross-sections fully occluded with atherosclerotic plaques, respectively) and correspondingly lower proportions of coronary artery cross sections that were devoid of atherosclerotic plaques (48.5% ± 2.6% vs. 80.6% ± 1.5%, respectively) compared to the 26 w.o. *SR-B1*^*KO/KO*^ mice that had been fed the HFCC diet for 12 weeks (Fig 2E). This demonstrates that *SR-B1*^*KO/KO*^ mice are more susceptible to HFCC diet-induced coronary artery atherosclerosis with older age.

### Older age increased VCAM 1 1evels and platelet accumulation in coronary arteries of HFCC diet-fed *SR-B1*^*KO/KO*^ mice

To investigate potential mechanisms underlying the increased susceptibility to HFCC diet-induced coronary artery atherosclerosis observed in 52 w.o. *SR-B1*^*KO/KO*^ mice, we examined levels VCAM-1 in non-atherosclerotic coronary arteries, since induction of VCAM-1 protein the intima of arteries plays a major role in the initiation of atherosclerosis [3–6]. Representative images of VCAM-1 immunofluorescence (Fig 2F-2J; corresponding oil red O/hematoxylin images shown in Fig 2K-2O) and quantification of VCAM-1 staining (Fig 2P) revealed that VCAM-1 levels were increased in non-atherosclerotic coronary artery cross sections from HFCC diet-fed 52 w.o. *SR-B1*^*KO/KO*^ mice that had been fed the HFCC diet for 12 weeks compared to all other experimental groups, including 52 w.o. *SR-B1*^*KO/KO*^ mice that had been maintained on the normal diet, 26 w.o. *SR-B1*^*KO/KO*^ mice that had been fed the HFCC for 12 weeks, and both the 26 and 52 w.o. C57BL/6J mice that had been fed the HFCC diet for 12 weeks. In contrast, the plasma levels of soluble VCAM-1 or ICAM-1 were not different between the 26 w.o. and 52 w.o. *SR-B1*^*KO/KO*^ or C57BL/6J mice that had been fed the HFCC diet for 12 weeks, although levels were increased in the 52 w.o. *SR-B1*^*KO/KO*^ mice that had been fed the HFCC diet compared to those fed the normal diet (S1 Fig.). This suggests advanced age did not cause a general increase in HFCC-diet induction of VCAM-1 but that coronary arteries may have been selectively impacted.

Platelets are known to play important roles not only in thrombosis upon plaque rupture [7,54], but also in early stages of atherosclerosis by adhering to activated endothelium and releasing chemokines that facilitate leukocyte adhesion to the artery wall, and secreting inflammatory factors that can activate endothelial cells [55–57]. We have previously shown that normal diet-fed *SR-B1*^*KO/KO*^*ApoE*^*KO/KO*^ mice and HFCC diet-fed *SR-B1*^*KO/KO*^*Ldlr*^*KO/KO*^ mice (which lack *SR-B1* together with either *ApoE* or *Ldlr* gene expression) exhibited evidence of platelet accumulation in atherosclerotic coronary arteries, suggesting possible contributions of platelets in driving atherosclerosis development and/or thrombus formation [17,45,50]. Therefore, we examined whether platelet accumulation would be evident in the coronary arteries of our mice via platelet CD41 immunostaining. Since we quantified CD41 staining in atherosclerotic coronary arteries, we only analyzed HFCC diet-fed 26 w.o. and 52 w.o. SR-B1 KO mice, since the HFCC diet-fed C57BL/6J mice and normal diet-fed *SR-B1*^*KO/KO*^ mice developed little-to-no coronary artery atherosclerosis. This analysis (Fig 2Q-2U) revealed that the 52 w.o. *SR-B1*^*KO/KO*^ mice that had been fed the HFCC diet for 12 weeks had significantly greater proportions of atherosclerotic coronary arteries that were positive for CD41 compared to the 26 w.o. *SR-B1*^*KO/KO*^ mice that had been fed the HFCC diet for the same duration (60.2% ± 4.3% vs. 37.8% ± 4.1%, respectively, P = 0.001). Therefore, older age was accompanied by increased accumulation of platelets in atherosclerotic coronary arteries of HFCC diet-fed *SR-B1*^*KO/KO*^ mice, suggesting that the coronary arteries in HFCC diet-fed 52 w.o. *SR-B1*^*KO/KO*^ mice may be more susceptible to platelet-driven atherosclerosis development and/or thrombosis.

### Older age increased myocardial fibrosis levels in HFCC diet-fed *SR-B1*^*KO/KO*^ mice

**Fig 3:**
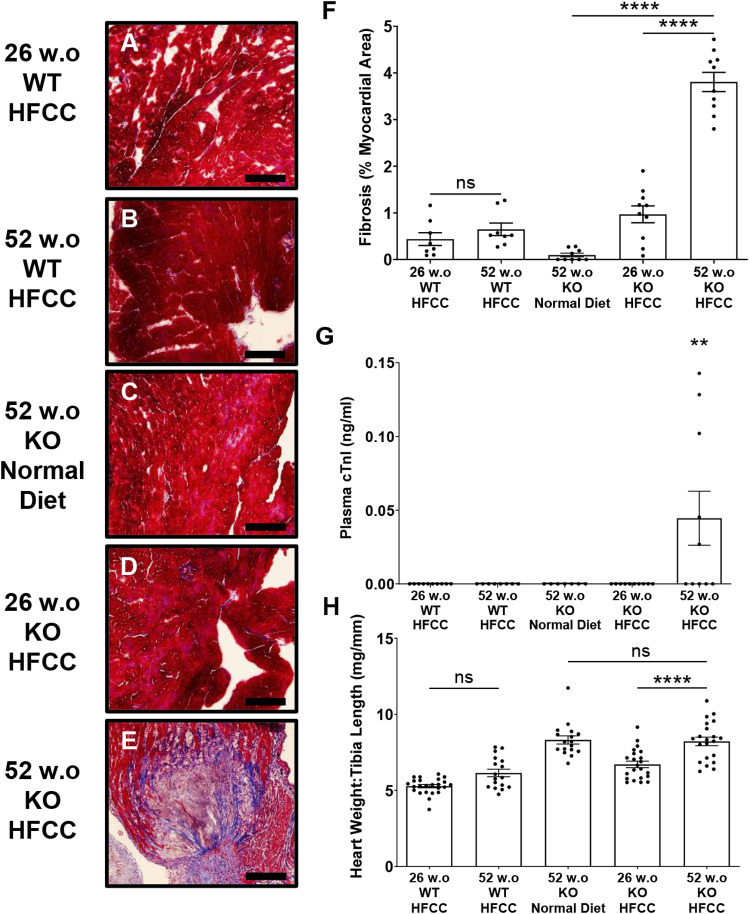
Increased myocardial fibrosis and plasma cardiac troponin I levels in older aged HFCC diet-fed *SR-B1*^*KO/KO*^ mice. Masson’s trichrome-staining of cardiac sections from (A) 26 w.o. and (B) 52 w.o. C57BL/6J (WT) mice that had been fed the HFCC diet for 12 wks; and *SR-B1*^*KO/KO*^ mice (KO) that were **(C)** 52 w.o. and maintained on a normal diet, or **(D)** 26 w.o. or **(E)** 52 w.o. and had been fed the HFCC diet for 12 weeks. Representative images are shown. Collagen stains blue/purple and intact myocardium stains red. Scale bar = 200 μm. **(F)** Percentage area of fibrotic tissue relative to heart cross-sectional area [45] (n = 8, 8, 9, 10, 10 mice/group). **(G)** Plasma cardiac troponin I levels (n = 10, 8, 7, 10, 10 mice/group). **(H)** Heart-to-tibia length ratios (n = 24, 15, 13, 22, 21 mice/group). Data points represent individual mice, bars show means and error bars show standard errors of the mean. Data were analyzed by one-way ANOVA and Tukey’s post-hoc test. ****P < 0.0001, **P < 0.01, ns = not statistically significantly different.

The increased extent of coronary artery atherosclerosis prompted us to examine the extent of myocardial fibrosis in the 52 versus 26 w.o. *SR-B1*^*KO/KO*^ mice that had been fed the HFCC diet for 12 weeks, as well as control similarly aged C57BL/6J mice fed the HFCC diet for an equivalent duration and control 52 w.o. *SR-B1*^*KO/KO*^ mice maintained on the normal diet. Masson’s trichrome staining of myocardial tissue (Fig 3A-3E) revealed substantially higher levels of cardiac fibrosis (blue/purple staining of collagen rich areas, Fig 3E, 3F) in sections from hearts from the 52 w.o. *SR-B1*^*KO/KO*^ mice that had been fed the HFCC diet for 12 weeks compared to the levels detected in the 26 w.o. *SR-B1*^*KO/KO*^ mice (Fig 3D, 3F; 3.8% ± 0.2% vs. 1.0% ± 0.2%, respectively, P < 0.0001) which were not significantly different from the low levels of fibrosis detected in either 26 or 52 w.o. C57BL/6J WT mice fed the HFCC diet for 12 weeks (Fig 3A,3B, 3F). Normal diet fed 52-week-old *SR-B1*^*KO/KO*^ mice exhibited little detectable myocardial fibrosis (Fig 3C, 3F). The levels of cardiac fibrosis parallel the levels of coronary artery atherosclerosis. Additionally, plasma cardiac troponin I levels, a marker of cardiac damage, were significantly elevated in 52 w.o. *SR-B1*^*KO/KO*^ mice the HFCC diet for 12 weeks, compared to all other groups, including 26 w.o. *SR-B1*^*KO/KO*^ mice fed the HFCC diet, in which plasma cardiac troponin I was not detectable (Fig 3G, P = 0.01). We also examined heart weight-to-tibia length ratios, an indicator of myocardial enlargement, and found that this increased with age but not with HFCC diet feeding in the *SR-B1*^*KO/KO*^ mice, suggesting that the increased coronary artery atherosclerosis and myocardial fibrosis did not induce cardiomegaly (Fig 3H). Nevertheless, older age increased myocardial fibrosis and levels of cardiac damage in *SR-B1*^*KO/KO*^ mice fed the HFCC diet.

### Older age reduced survival of HFCC diet-fed *SR-B1*^*KO/KO*^ mice

**Fig 4:**
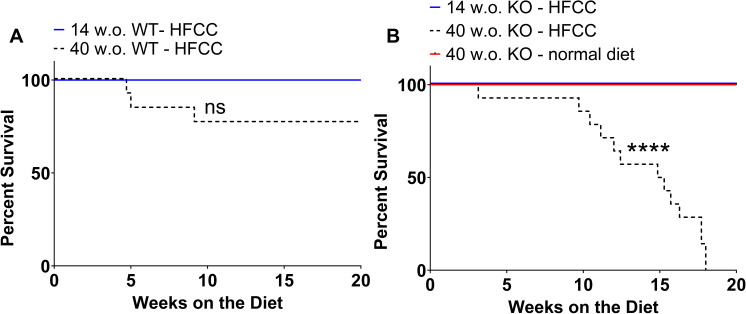
Reduced survival upon HFCC diet-feeding of older SR-B1^KO/KO^ mice. Kaplan-Meier survival curves for **(A)** 14-week-old (w.o.) (solid blue line, n = 13) and 40 w.o. (dotted black line, n = 13) C57BL/6J (WT) mice and **(B)** 14 w.o. (solid blue line, n = 14) and 40 w.o. (dotted black line, n = 14) *SR-B1*^*KO/KO*^ (KO) mice fed the HFCC diet for up to 20 weeks and 40 w.o. *SR-B1*^*KO/KO*^ (KO) mice maintained on normal diet for an additional 20 weeks (solid red line, n = 12). Mice were monitored until they reached cardiac endpoint or the end of 20 weeks of diet feeding, at which point they were humanely euthanized. Data were analyzed by the Mantel-Cox log-rank test. ****P < 0.0001; ns = not statistically significantly different.

To investigate whether the observed augmentation of coronary artery atherosclerosis, myocardial damage and myocardial fibrosis in the older compared to the younger *SR-B1*^*KO/KO*^ mice might be accompanied by reduced survival to humane endpoint, we fed both *SR-B1*^*KO/KO*^ and control C57BL/6J WT mice the HFCC diet for 20 weeks beginning when mice were either 14 or 40 weeks of age. All of the 14 w.o. C57BL/6J mice (n = 13) and 10 out of 13 40 w.o. C57BL/6J mice survived up to 20 weeks of HFCC diet-feeding, while 3 of the 13 mice died unexpectedly (Fig 4A). It is not clear why 3 of the 40 w.o. C57BL/6J mice died. However, since C57BL/6J have been shown to develop hepatotoxicity when fed a high-fat diets [58–60], we examined whether older age affected levels of plasma alanine aminotransferase (ALT) and aspartate aminotransferase (AST), both markers of liver damage [60]. Although there were no significant differences in ALT and AST levels between 14 w.o. and 40 w.o. C57BL/6J mice fed the HFCC diet for 12 weeks (26 w.o. and 52 w.o., respectively) (S2 Fig A and B), trends towards increased levels of both were observed in 52 w.o. compared to 26 w.o. C57BL/6J mice fed the HFCC diet for 12 weeks. We cannot confirm if the 3 older aged C57BL/6J mice that died during the HFCC diet feeding period exhibited elevated ALT and AST levels indicative of liver damage compared to those that survived up to 20 weeks of HFCC diet feeding, although this seems like a possible explanation. Nonetheless, there were no significant differences in survival between 14 w.o. and 40 w.o. C57BL/6J mice fed the HFCC diet. All the 14 w.o. *SR-B1*^*KO/KO*^ mice (n = 14) survived up to 20 weeks of HFCC diet-feeding (Fig 4B). Interestingly, all of the 40 w.o. *SR-B1*^*KO/KO*^ mice (n = 14) fed the HFCC diet exhibited the constellation of symptoms (ruffled coat, hunched posture, lethargy, and labored breathing) triggering humane endpoint, with an average onset of symptoms of 14 weeks after initiation of HFCC diet feeding (Fig 4B). To determine whether the reduced survival in *SR-B1*^*KO/KO*^ mice was diet-dependent, a separate cohort of 40 w.o. *SR-B1*^*KO/KO*^ mice were left on a normal diet for up to 20 additional weeks (n = 12), and all the mice survived without exhibiting signs triggering humane endpoint (Fig 4B). Therefore, older age significantly reduced the symptom-free survival of HFCC diet-fed *SR-B1*^*KO/KO*^ mice.

### Older age increased circulating proinflammatory cytokine levels but not lipid levels in HFCC diet-fed *SR-B1*^*KO/KO*^ mice

**Fig 5:**
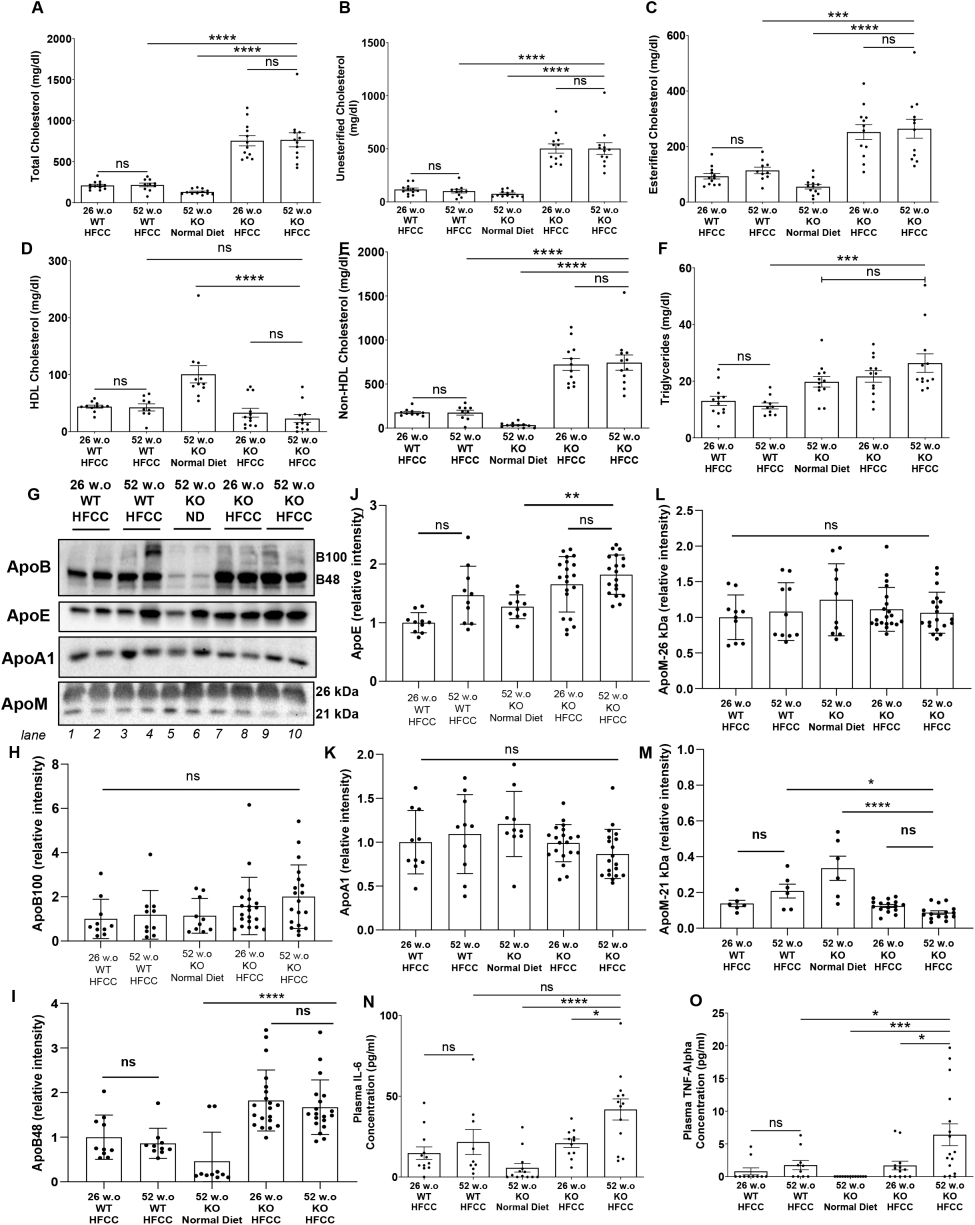
Increased interleukin 6 and tumor necrosis factor **α** **but not cholesterol or triglyceride levels in older aged HFCC diet-fed *SR-B1***^***KO/KO***^ **mice.** Plasma was collected from 26 and 52 w.o. C57BL/6J (WT) mice that had been fed the HFCC diet for 12 wks and *SR-B1*^*KO/KO*^ mice (KO) that were 52 w.o. and maintained on a normal diet, or were 26 or 52 w.o. and had been fed the HFCC diet for 12 weeks. Plasma levels of **(A)** total cholesterol, **(B)** unesterified and **(C)** esterified cholesterol (calculated as the difference between total and free cholesterol), **(D)** HDL cholesterol (phosphotungstate-magnesium non-precipitable), **(E)** non-HDL cholesterol (calculated as the difference between total and HDL-cholesterol) and **(F)** triglycerides (n = 12, 10, 12, 12, 12 mice/group for **A-C** and **F**; n = 11, 9, 12, 12, 11 mice/group for **D** and **E**). **(G)** Immunoblotting (representative blots shown; n = 2/group) and **(H-M)** quantification of relative band intensities for **(H)** apoB100, **(I)** apoB48, **(J)** apoE, **(K)** ApoA1 and **(L)** the major (26 kDa band) and **(M)** the minor (21 kDa band) of ApoM. Band intensities for apoB100 and apoB48 were quantified using different exposures of the same blot. Band intensities for apoB100, apoB48, apoE and apoA1 are expressed relative to the average intensity of the 14w.o. WT-HFCC diet group (lanes 1 and 2). Band intensities for the apoM 26 kDa and 21 kDa bands were quantified using the same blot and are expressed relative to the intensity of the 26 kDa band of the 14w.o. WT-HFCC diet group (lanes 1 and 2). Group sizes were n = 10,10,10,20,19 mice/group for panels **H-L** and n = 6,6,6,16,15 mice/group for panel **M**. Plasma levels of **(N)** interleukin 6 (IL-6) (n = 12, 9, 13, 11, 13 mice/group) and **(O)** tumor necrosis factor α (TNFα) (n = 10, 10, 13, 13, 16 mice/group). Data points represent individual mice, bars show means and error bars show standard errors of the mean. All data were analyzed by one-way ANOVA and Tukey’s post-hoc test. ****P < 0.0001, ***P < 0.001, **P < 0.01, *P < 0.05, ns = not statistically significantly different.

Consistent with previous findings, the HFCC diet-fed *SR-B1*^*KO/KO*^ mice exhibited higher total and free cholesterol levels than HFCC diet-fed C57BL/6J WT mice and higher total, free and esterified cholesterol levels than normal diet-fed *SR-B1*^*KO/KO*^ mice (Fig 5A, 5B and 5C). In contrast, the 26 and 52 w.o. HFCC diet-fed *SR-B1*^*KO/KO*^ mice exhibited lower levels of cholesterol associated with phosphotungstate-magnesium non-precipitable (normal) HDL (Fig 5D) and higher levels of cholesterol associated with precipitable lipoproteins (non-HDL, Fig 5E) compared to normal diet-fed 52 w.o. *SR-B1*^*KO/KO*^ mice. Nevertheless, there were no statistically significant differences in any of these parameters between HFCC diet-fed 26 w.o. and 52 w.o. *SR-B1*^*KO/KO*^ mice (Fig 5A-5E). Plasma triglyceride levels were higher in *SR-B1*^*KO/KO*^ than in C57BL/6J WT mice but were not different between 26 w.o. or 52 w.o. mice that had been fed the HFCC diet for 12 weeks or between 52 w.o. *SR-B1*^*KO/KO*^ mice fed the HFCC diet or maintained on the normal diet (Fig 5F). Analyses of apolipoproteins (Fig 5G) revealed no statistically significant differences in apoB100, but increased apoB48 in HFCC diet-fed 26 w.o. and 52 w.o. *SR-B1*^*KO/KO*^ mice compared to HFCC diet fed 26 w.o. and 52 w.o. C57BL/67J mice and 52 w.o. *SR-B1*^*KO/KO*^ mice fed the normal diet (Fig 5H, 5I), and increased levels of apoE in HFCC diet-fed 26 w.o. and 52 w.o. compared to normal diet fed 52 w.o. *SR-B1*^*KO/KO*^ mice (Fig 5J). No statistically significant differences in relative levels of apoA1 (Fig 5K) or the major 26 kDa band of apoM (Fig 5L) were observed; however the levels of the minor 21 kDa apoM band were reduced in HFCC diet fed 26 w.o. and 52 w.o. compared to normal diet fed 52 w.o. *SR-B1*^*KO/KO*^ mice (Fig 5M). No statistically significant differences were observed in the relative levels of any of these apolipoproteins in plasma from 26 w.o. versus 52 w.o. *SR-B1*^*KO/KO*^ mice that had been fed the HFCC diet for 12 weeks (Fig 5G-5M).

There were marked increases in plasma levels of the pro-inflammatory cytokines IL-6 (Fig 5E) and TNF-α (Fig 5F) in 52 w.o. compared to 26 w.o. *SR-B1*^*KO/KO*^ mice that had been fed the HFCC diet for 12 weeks and compared to 52 w.o. *SR-B1*^*KO/KO*^ mice maintained on normal diet. The levels of these cytokines were not different between 52 and 26 w.o. C57BL/6J WT mice that had been fed the HFCC diet for 12 weeks. This suggests that older age increases the susceptibility of *SR-B1*^*KO/KO*^ mice to HFCC-diet induced inflammation.

### Older age increased circulating neutrophils in HFCC diet-fed *SR-B1*^*KO/KO*^ mice

**Fig 6:**
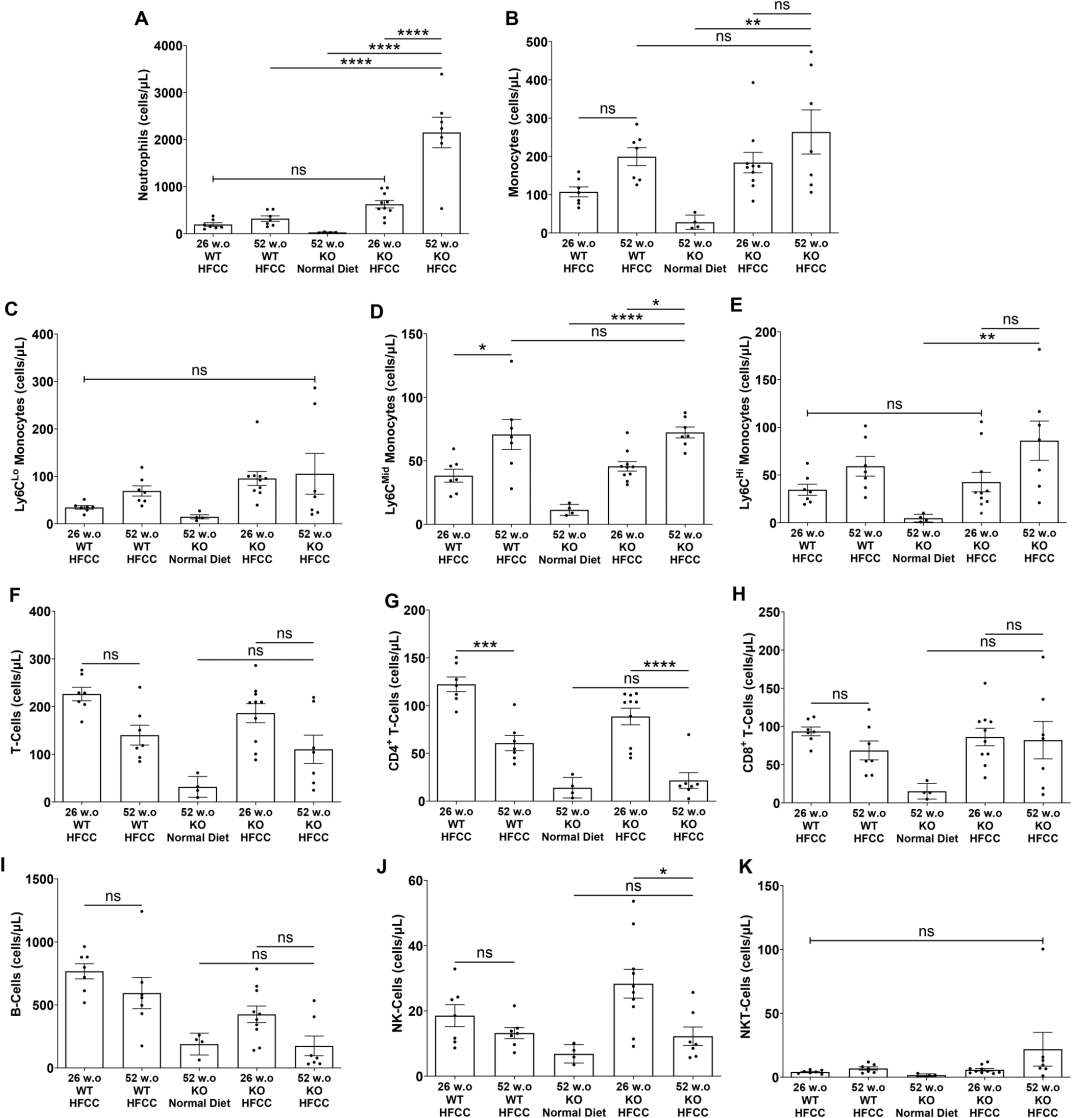
Flow cytometry analysis of blood leukocytes. Blood collected from 26 and 52 w.o. C57BL/6J mice (WT) that had been fed the HFCC diet for 12 wks, and from 26 or 52 w.o. *SR-B1*^*KO/KO*^ (KO) mice that had either been maintained on the normal diet or fed the HFCC diet for 12 wks as indicated. **(A)** Neutrophils. **(B)** Monocytes. **(C)** Ly6C^lo^, **(D)** Ly6C^mid^ and **(E)** Ly6C^hi^ monocytes. **(F)** Total, **(G)** CD4^+^ and **(H)** CD8^+^ T-cells. **(I)** B-cells. **(J)** Natural killer (NK) cells. **(K)** Natural killer T (NKT) cells. N = 7, 7, 4, 10, 7 mice per group for all flow cytometry analyses. Data points represent individual mice, bars show means and error bars show standard errors of the mean. Statistical analysis was by one-way ANOVA and Tukey’s post-hoc test. ****P < 0.0001, ***P < 0.001, **P < 0.01, *P < 0.05, ns = not statistically significantly different.

Flow cytometry analyses of leukocyte populations revealed significant increases in the numbers of circulating neutrophils with the *SR-B1*^*KO/KO*^ genotype in HFCC-diet fed 52 w.o. mice and with age in *SR-B1*^*KO/KO*^ mice with substantially higher levels detected in 52 w.o. compared to 26 w.o. *SR-B1*^*KO/KO*^ mice after HFCC-diet feeding for 12 weeks (Fig 6A). HFCC diet feeding was accompanied by increases in total monocytes and in Ly6CLo, Mid, Hi and monocytes in 52 w.o. *SR-B1*^*KO/KO*^ mice and trends (which did not reach statistical significance) towards increases in total and Ly6Chi monocytes in *SR-B1*^*KO/KO*^ versus C57BL/6J mice and between 52 w.o. and 26 w.o. *SR-B1*^*KO/KO*^ mice (Fig 6B-6E). We also noted trends towards reduced levels of CD4+ and total T cells, B cells and NK cells but not CD8+ or NK T cells in 52 w.o. compared to 26 w.o. *SR-B1*^*KO/KO*^ mice that had been fed the HFCC diet for 12 weeks (Fig 6F-6K). These observations suggest that older age was associated with altered circulating levels of a variety of inflammatory and immune cells which could have contributed to the age-dependent increased susceptibility of SR-B1 KO mice to coronary artery atherosclerosis.

**Fig 7:**
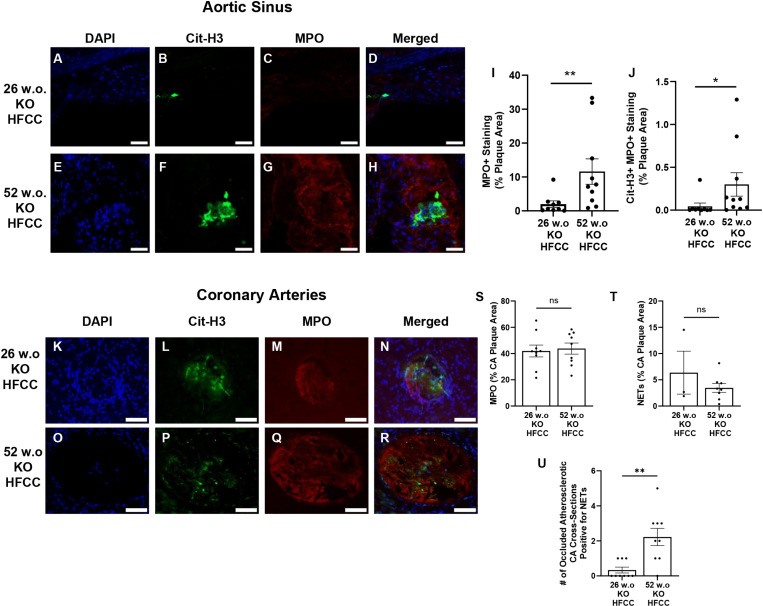
Immunofluorescence staining for MPO and Cit-H3 in atherosclerotic plaques. Representative images (**A-H** and **K-R**) of aortic sinus (**A-H**) and coronary artery (**K-R**) atherosclerotic plaques from 26 w.o. (**A-D** and **K-N**) and 52 w.o. (**E-H** and **O-R**) *SR-B1*^*KO/KO*^ mice that had been fed the HFCC diet for 12 weeks. Quantification of the extent of **(I)** MPO and **(J)** Cit-H3 and MPO co-staining in aortic sinus atherosclerotic plaques and **(S)** MPO and **(T)** Cit-H3 and MPO co-staining (representing NETs) in coronary atherosclerotic plaques. **(U)** Numbers of occluded atherosclerotic coronary artery cross sections that were positive for Cit-H3 and MPO co-staining (representing NETs). Each data point represents a different mouse. Bars represent means and error bars represent standard errors of the mean. Data was analyzed by the Mann-Whitney Rank Sum test. ***P < 0.001, **P < 0.01, * P < 0.05, ns = not statistically significantly different.

To explore the contribution of neutrophils to aortic sinus and coronary artery atherosclerosis in these mice, we stained aortic sinus and myocardial sections for the neutrophil marker, MPO as well as for Cit-H3, a marker of neutrophil extracellular traps (NETs) [20]. No staining was observed in any of the cross sections of arteries that did not contain atherosclerotic plaques (including the HFCC diet fed 26 w.o. and 52 w.o. C57BL/6J mice and the 52 w.o. *SR-B1*^*KO/KO*^ mice that had been fed the normal diet); therefore, only aortic sinus and coronary artery sections with atherosclerotic plaques from the HFCC diet-fed 26 w.o. and 52 w.o. *SR-B1*^*KO/KO*^ mice were analyzed (Fig 7). This revealed increased MPO staining and increased evidence of NETs (Cit-H3+ and MPO+ staining) in atherosclerotic plaques in the aortic sinuses of 52 w.o. *SR-B1*^*KO/KO*^ compared to 26 w.o. *SR-B1*^*KO/KO*^ mice when both were fed the HFCC diet for 12 weeks (Fig 7A-7J). MPO and NET staining were only observed in coronary artery cross sections that were completely occluded by atherosclerotic plaques (Fig 7K-7R). Analysis of these sections revealed no differences in the extent of MPO staining within the atherosclerotic plaques between 52 and 26 w.o. *SR-B1*^*KO/KO*^ mice that had been fed the HFCC diet for 12 weeks (Fig 7S). Cit-H3 was detected in only 3 occluded coronary arteries from 26 w.o. *SR-B1*^*KO/KO*^ mice that had been fed the HFCC diet for 12 weeks; however there was no apparent difference in the extent of NET staining in the occluded coronary artery sections analyzed from the 52 and 26 w.o. *SR-B1*^*KO/KO*^ mice that had been fed the HFCC diet for 12 weeks (Fig 7T). However the number of occluded coronary artery cross sections that were positive for NET staining was increased in the 52 w.o. versus the 26 w.o. *SR-B1*^*KO/KO*^ mice that had been fed the HFCC diet for 12 weeks (Fig 7U).

## Discussion

In this study, we demonstrate *SR-B1*^*KO/KO*^ mice (in the absence of KO mutations in either *ApoE* or *Ldlr*) exhibit increased susceptibility to atherosclerosis development in coronary arteries and cardiac fibrosis with advanced age. Our data suggest that this was likely driven by a combination of increased VCAM-1 and platelet accumulation in coronary arteries and increased systemic inflammation. The significant increase in coronary artery atherosclerosis and myocardial infarction, as indicated by increased plasma cardiac troponin I and increased cardiac fibrosis, in HFCC diet-fed older aged *SR-B1*^*KO/KO*^ mice likely explains their reduced survival.

Surprisingly, there were no differences in the sizes of atherosclerotic plaques within the aortic sinus of 52 w.o. compared to 26 w.o. *SR-B1*^*KO/KO*^ mice when they were fed the HFCC diet for 12 weeks. One possible explanation might be that atherosclerotic plaque growth in the aortic sinus may have reached a maximum in both groups of mice by the end of the 12 week HFCC diet feeding period, masking any effects of older age on the susceptibility of *SR-B1*^*KO/KO*^ mice to development of atherosclerosis in the aortic sinus induced by the HFCC diet. However, we have previously reported [37] that feeding young (10-week old) female *SR-B1*^*KO/KO*^ mice the HFCC diet for 20 weeks resulted in average plaque sizes (~ 400,000 μm^2^) that were double those reported here-in when equivalent mice were fed the HFCC diet for 12 weeks, indicating that under the conditions employed in this study, atherosclerotic plaques did not reach a maximum size in the aortic sinus. Instead, in the context of our prior studies, this suggests that older age did not increase the susceptibility to aortic sinus atherosclerosis, at least as measured by cross-sectional areas. This appears to differ from other studies of the effects of age on atherosclerosis, in which either *ApoE*^*KO/KO*^ or *Ldlr*^*KO/KO*^ mice have been used, where increased atherosclerosis has been detected in aortas of older aged mice [38,39]. However, both *ApoE*^*KO/KO*^ and *Ldlr*^*KO/KO*^ mice develop atherosclerosis spontaneously when they are fed normal diets [10,11,40], making it difficult to differentiate the effects of age on the susceptibility to develop atherosclerosis from the effects of time over which atherosclerotic plaques have been allowed to develop. In contrast, *SR-B1*^*KO/KO*^ mice appear to develop no detectable atherosclerosis in their aortic sinus when fed a normal diet, even up to 52 weeks of age, suggesting that atherosclerosis development in these mice is entirely dependent on atherogenic diet feeding. This allows for a more precise evaluation of the susceptibility of older versus younger mice to develop atherosclerosis because of the absence of spontaneous atherosclerotic plaque development.

Older aged *SR-B1*^*KO/KO*^ mice did not exhibit altered plasma cholesterol or triglyceride levels when compared to younger aged *SR-B1*^*KO/KO*^ mice when they were fed the HFCC diet, demonstrating that the increased coronary artery atherosclerosis was not driven by increased plasma cholesterol in the older versus the younger *SR-B1*^*KO/KO*^ mice. Instead, older aged HFCC diet-fed *SR-B1*^*KO/KO*^ mice had increased blood neutrophil and trends towards increased blood monocyte levels, levels of IL-6 and TNFα in plasma and increased levels of VCAM1 on coronary arteries compared to younger aged *SR-B1*^*KO/KO*^ mice fed the same diet. The increased levels of neutrophils in blood in older aged mice was also reflected by the detection of increased levels of the neutrophil marker MPO as well as citrullinated histone H3, a marker of NETs in atherosclerotic plaques in the aortic sinuses and increased numbers of atherosclerotic coronary artery cross sections exhibiting NETs in the older aged HFCC diet fed *SR-B1*^*KO/KO*^ mice. Similar detection of neutrophil including NET markers in atherosclerotic plaques has recently been reported in a related mouse model of coronary heart disease and stroke, with a mutation impacting SR-B1, the *SR-B1-delta CT*/*Ldlr KO* mouse fed a high fat diet [20]. The increased levels of these markers in atherosclerotic plaques in the aortic sinuses of older aged HFCC diet fed *SR-B1*^*KO/KO*^ mice despite the similar plaque sizes and levels of VCAM1, suggests that advanced age may nevertheless increase plaque inflammation and composition in the aortic sinus as well as increasing the burden of coronary artery atherosclerosis in these mice.

The increased plasma TNFα and IL-6 levels observed in older compared to younger aged SR-B1^KO/KO^ mice fed the HFCC diet is consistent with previous reports of increased levels of these cytokines with advanced age, a process thought to contribute to the increased burden of chronic diseases, such as cardiovascular disease, with age [61–63]. This suggests that the increased susceptibility of older *SR-B1*^*KO/KO*^ mice to HFCC diet-induced coronary artery atherosclerosis is the result of increased inflammation and recruitment of leukocytes to coronary arteries. We previously reported similar findings (increased coronary artery VCAM-1, circulating monocyte levels, and coronary artery atherosclerosis and cardiac fibrosis) when comparing *SR-B1*^*KO/KO*^*Ldlr*^*KO/KO*^ mice with *Ldlr*^*KO/KO*^ mice with intact SR-B1 genes, when both were fed the HFCC diets [17]. Since VCAM-1 on endothelial cells plays a major role in initiating and driving atherosclerosis development, it is likely that this increased VCAM-1 expression in coronary arteries at least in part explains the increased coronary artery atherosclerosis. This also suggests that older age in HFCC diet-fed *SR-B1*^*KO/KO*^ mice may increase the susceptibility of coronary arteries to endothelial activation, which in turn increases the susceptibility to leukocyte recruitment and initiation of atherosclerosis.

We also previously reported that atherogenic diet-fed *SR-B1*^*KO/KO*^*Ldlr*^*KO/KO*^ mice showed evidence of platelet accumulation in atherosclerotic coronary arteries [17,45,50]. HFCC diet-fed 52 w.o. *SR-B1*^*KO/KO*^ mice exhibited increased proportions of atherosclerotic coronary arteries that were positive for platelets compared to HFCC diet-fed 26 w.o. *SR-B1*^*KO/KO*^ mice. Considering the role of platelets in both the early stages of atherosclerosis as well as thrombosis [7,54–57], the increased platelet accumulation in atherosclerotic coronary arteries may in part explain the increased coronary artery atherosclerosis and/or cardiac fibrosis seen in HFCC diet-fed 52 w.o. *SR-B1*^*KO/KO*^ mice. However, it is difficult to determine whether the platelet accumulation occurred during atherosclerosis development, or was a consequence of plaque rupture and subsequent thrombosis.

### Limitations

It is important to consider the limitations of this study. Firstly, only female mice were included in this study. However, in findings to be reported elsewhere, we observe similar effects of age on the extent of HFCC-diet induced atherosclerosis in coronary arteries and aortic sinuses of male *SR-B1*^*KO/KO*^ mice. Secondly, the atherosclerotic plaques in the coronary arteries of these mice do not appear to mirror the complexity of atherosclerotic plaques in human coronary arteries. One reason for this may be the much smaller size and different hemodynamic properties of coronary arteries in mice compared to those in humans. Thirdly, we did not investigate other vascular beds such as carotid [20] or cerebral arteries (relevant to stroke), or peripheral arteries, such as the descending aorta or femoral arteries (relevant to peripheral artery disease). Therefore we have not investigated the extent to which advanced age impacts susceptibility to diet induced atherosclerosis in other arteries relevant to human disease. Fourth, SR-B1 in liver plays a key role in driving HDL dependent reverse cholesterol transport, which is impaired in *SR-B1*^*KO/KO*^ mice. Therefore, age dependent impacts on reverse cholesterol transport cannot be studied using this model. Finally, we have not yet carried out studies to probe the mechanisms of the impacts of advanced age on atherosclerosis in coronary atherosclerosis and the aortic sinus. Future studies to probe mechanisms will include blockade of VCAM-1, TNFα or IL-6 or depletion of neutrophils to examine the extent to which increased VCAM1, TNFα, IL-6 and neutrophilia with advanced age drives the increased burden of coronary artery atherosclerosis.

## Conclusions

In summary, HFCC diet-fed female *SR-B1*^*KO/KO*^ mice develop occlusive coronary artery atherothrombosis and cardiac fibrosis and a plasma marker of myocardial infarction in an age-dependent manner. The extent to which age impacts coronary artery atherothrombosis and markers of myocardial infarction in other mouse models commonly used to study atherosclerosis such as *ApoE*^*KO/KO*^ or *Ldlr*^*KO/KO*^ mice remains to be determined but is complicated by the spontaneous nature of atherosclerosis development in those models, in which plaques develop over time even when mice are fed diets low in fat content and devoid of cholesterol. Therefore, female *SR-B1*^*KO/KO*^ mice, in which atherosclerosis development appears to be entirely dependent on atherogenic diet feeding, may prove to be a useful mouse model to dissect the pathways that are involved in age-dependent CAD development, particularly in the context of preserved LDL and VLDL clearance pathways (due to intact *Ldlr* and *ApoE* expression). Therefore, they may be particularly useful for analyzing the impacts of age of therapeutics such as statins and/or PCSK9 inhibitors that target these pathways and in the context of coronary heart disease. Further analyses of aged *SR-B1*^*KO/KO*^ mice may help elucidate the factors that contribute to age-related development of coronary artery disease and contribute to identifying novel therapeutic targets to treat cardiovascular disease in the aged population.

## Supporting information

S1 Supplemental Figs 1-2. (PDF)

S2 Raw data for graphs. (XLSX)

## Nonstandard Abbreviations and Acronyms

ALT: alanine aminotransferase
AST: aspartate aminotransferase
ApoE: apolipoprotein E
CAD: coronary artery disease
ICAM-1: intercellular adhesion molecule 1
IL-6: interleukin 6
LDLR: low-density lipoprotein receptor
SR-B1: scavenger receptor class B type I
TNFα: tumour necrosis factor alpha
VCAM-1: vascular cell adhesion molecule 1
