# Supplementary material for: Scavenger receptor class B type I knockout mice develop extensive diet-induced coronary artery atherosclerosis in an age-dependent manner
Source: PLoS One. 2025 May 22;20(5):e0318118. doi: 10.1371/journal.pone.0318118 (PMC12097598; doi:10.1371/journal.pone.0318118)
Supplement: S1 — (PDF) [file pone.0318118.s001.pdf]

B

A

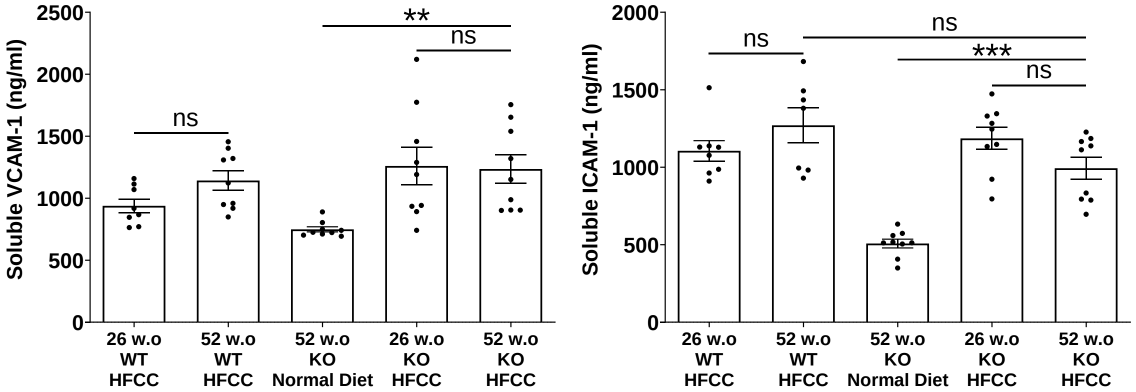

Supplementary Figure I. Soluble vascular cell adhesion molecule 1 (VCAM-1) and intercellular adhesion molecule 1 (ICAM-1) levels in mice. **(A)** Soluble VCAM-1 and **(B)** Soluble ICAM-1 in plasma from 26 and 52 w.o. C57BL/6J mice (WT) that had been fed the HFCC diet for 12 wks, and from 26 or 52 w.o. *SR-B1*$^{KO/KO}$ (KO) mice that had either been maintained on the normal diet or fed the HFCC diet for 12 wks as indicated. Each data point represents an individual mouse. Bars represent means and error bars represent standard errors of the mean. All data were analyzed by one-way ANOVA and Tukey's post-hoc test. **P <0.01, ***P <0.001.

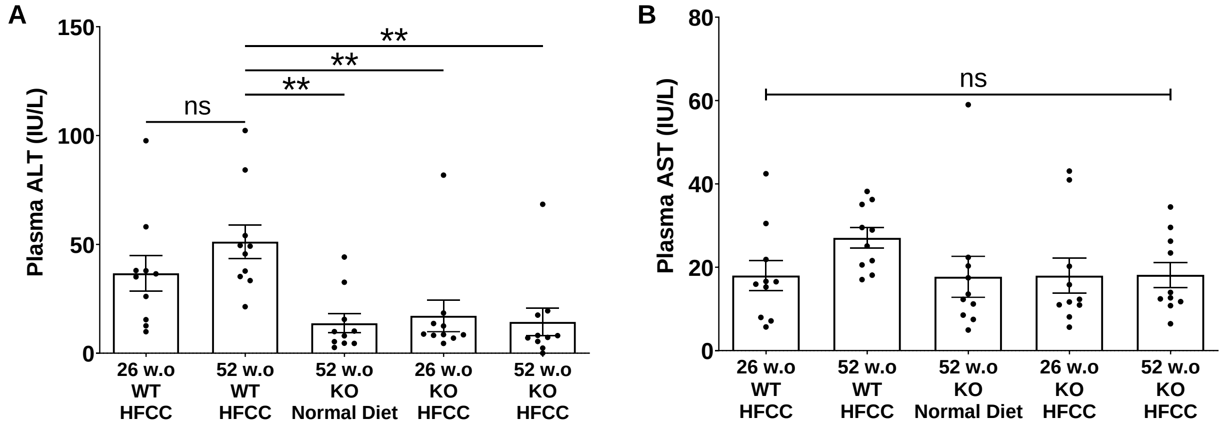

Supplementary Figure II. Plasma levels of alanine aminotransferase (ALT) and aspartate aminotransferase (AST). **(A)** ALT and **(B)** AST levels in plasma from 26 and 52 w.o. C57BL/6J mice (WT) that had been fed the HFCC diet for 12 wks, and from 26 or 52 w.o. *SR-B1*$^{KO/KO}$ (KO) mice that had either been maintained on the normal diet or fed the HFCC diet for 12 wks as indicated. Each data point represents an individual mouse. Bars represent means and error bars represent standard errors of the mean. All data were analyzed by one-way ANOVA and Tukey's post-hoc test. **P <0.01.
